# Use of deep sequencing data for routine analysis of HIV resistance in newly diagnosed patients

**DOI:** 10.7448/IAS.17.4.19748

**Published:** 2014-11-02

**Authors:** Jose-Angel Fernández-Caballero, Natalia Chueca, Marta Alvarez, Dimitri Gonzalez, Federico García

**Affiliations:** 1 Microbiology & Infectious Diseases, HU San Cecilio, Granada, Spain; 2 Bioinformatics, ABL Spain, Barcelona, Spain

## Abstract

**Introduction:**

Use of deep sequencing is becoming a critical tool in clinical virology, with an important impact in the HIV field for routine diagnostic purposes. Here, we present the comparison of deep and Sanger sequencing in newly diagnosed HIV patients, and the use of DeepChek v1.3 & VisibleChek for their interpretation and integration with virological and clinical data.

**Patients and Methods:**

Plasma samples from 88 newly diagnosed HIV-1-infected patients were included in the study. Median age (IQR) was 37 (27–47), median CD4 count (IQR) was 387 (220–554), and 85% were males. Median Viral Load (Log, IQR) was 5.03 (4.51–5.53). Deep sequencing was obtained using a GS-Junior (Roche). Sequences were preprocessed with the 454 AVA software; aligned reads were uploaded into the DeepChek v1.3 system (ABL SA). Sanger sequences (Trugene), were uploaded in parallel. Stanford algorithm (version 7.0) resistance interpretation to first line drugs and all the mutations (score≥5) were analyzed. For deep sequencing, 1%, 5% and 10% thresholds were chosen for resistance interpretation.

**Results:**

Using VisibleChek for analysis, we were able to describe the detection of any mutation using Sanger in 37/88 patients, with a total number of 50 Stanford ≥5 mutations, K103N and E138A being the most prevalent (n=4). Using UDS-1%, we found 72/88 patients with at least one mutation (total of 206 Stanford ≥5 mutations). Using Sanger data, 9/88 patients (10.22%) showed any resistance to NNRTIs, while none showed resistance to NRTIs or PIs. Using UDS-10% increased resistance to NRTIs [3/88 (3.40%)], to NNRTIs 12/88 (13.63%), and to a lesser extent to PIs [1/88 (1.13%)]. Using UDS-5% increased resistance to NRTIs [4/88 (4.54%)] and to NNRTIs [12/88 (13.63%)], but not to PIs. Using UDS-1% increased resistance to all classes: NRTIs [14/88 (15.90%)], NNRTIs [26/88 (30.68%)], and PIs [6/88 (6.81].

**Conclusions:**

DeepChek and VisibleChek allow for an easy, reliable and rapid analysis of UDS data from HIV-1. Compared to Sanger data, UDS detected a higher number of resistance mutations. UDS with a 5 &10% threshold resulted in an increase in the number of patients with any degree of resistance mainly to NRTI, NNRTIs. Going down as low as 1% increased resistance to all classes. A correct definition of clinically relevant thresholds for the interpretation of minor variant detection for different classes of ARVs is needed.

